# Influenza Epidemiology and Vaccine Effectiveness Following Funded Influenza Vaccine in Queensland, Australia, 2022

**DOI:** 10.1111/irv.70007

**Published:** 2024-09-25

**Authors:** Ashish C. Shrestha, Emma Field, Dharshi Thangarajah, Ross Andrews, Robert S. Ware, Stephen B. Lambert

**Affiliations:** ^1^ Queensland Public Health and Scientific Services, Queensland Health Brisbane Queensland Australia; ^2^ National Centre for Epidemiology and Population Health Australian National University Canberra Australian Capital Territory Australia; ^3^ Department of Health and Aged Care Canberra Australian Capital Territory Australia; ^4^ Griffith Biostatistics Unit Griffith University Brisbane Queensland Australia; ^5^ National Centre for Immunisation Research and Surveillance Sydney New South Wales Australia

**Keywords:** Australia, flu, influenza, influenza vaccine, Queensland, vaccine effectiveness

## Abstract

**Background:**

In 2022, publicly funded influenza vaccine was made available to all residents of Queensland, Australia. This study compared influenza epidemiology in 2022 with previous years (2017–2021) and estimated influenza vaccine effectiveness (VE) during 2022.

**Methods:**

The study involved a descriptive analysis of influenza notifications and a case–control study to estimate VE. Cases were notifications of laboratory‐confirmed influenza, and controls were individuals who were test negative for COVID‐19. Cases and controls were matched on age, postcode and specimen collection date. VE against hospitalisation was investigated by matching hospitalised cases to controls. Conditional logistic regression models were adjusted for sex.

**Results:**

In 2022, Queensland experienced an early influenza season onset (April–May) and high case numbers (*n* = 45,311), compared to the previous 5 years (annual average: 29,364) and 2020–2021 (2020:6047; 2021:301) during the COVID‐19 pandemic. Adjusted VE (VE_ad_j) against laboratory‐confirmed influenza was 39% (95% confidence interval [CI]: 37–41), highest for children aged 30 months to < 5 years (61%, 95% CI: 49–70) and lowest for adults aged ≥ 65 years (24%, 95% CI: 17–30). VE_adj_ against influenza‐associated hospitalisation was 54% (95% CI: 48–59). Among children < 9 years of age, VE_adj_ against laboratory‐confirmed influenza (55%, 95% CI: 49–61) and hospitalisation (67%, 95% CI: 39–82) was higher in those who received a complete dose schedule.

**Conclusion:**

In Queensland, the 2022 influenza season started earlier than the previous 5 years. VE against influenza notifications varied across age groups. VE estimates against influenza‐associated hospitalisation were higher than those against laboratory‐confirmed influenza.

## 1 Background

Seasonal influenza epidemics infect ~20% of the world's population annually, causing 290,000–650,000 deaths [1]. Influenza vaccination is known to reduce the risk of severe disease among high‐risk populations and can prevent further transmission [2]. In Australia, laboratory‐confirmed influenza is a nationally notifiable condition [3]. The National Immunisation Program (NIP) funds influenza vaccines for populations at risk including children aged 6 months to < 5 years old, all Aboriginal and Torres Strait Islander people (aged ≥ 6 months), individuals aged 65 years and older and pregnant women [4]. Adjuvanted influenza vaccine was first funded for adults aged ≥ 65 years for the 2020 influenza season [5]. There were a smaller number of influenza cases during COVID‐19 pandemic years, 2020 (*n* = 21,338) and 2021 (*n* = 748) [6]. Consequently, 2022 was the first year with sufficiently large case numbers to assess the effectiveness of adjuvanted vaccine among older adults [6]. In Queensland, the state government funded free influenza vaccines for all residents who were not eligible for NIP‐funded vaccine from 24 May 2022 to 17 July 2022 [7]. Only quadrivalent vaccines were used in Australia in 2022 with each vaccine containing two influenza A subtypes (H3N2 and H1N1) and two influenza B lineages (Victoria and Yamagata lineages) [8].

There has been limited study on influenza vaccine effectiveness (VE) in Queensland, with a previous study reporting effectiveness for the < 5 years age group [9]. With this study, we aimed to understand the epidemiology of influenza and estimate VE across all ages following the introduction of the state government funded influenza vaccine program for all Queenslanders in 2022.

## 2 Methods

### 2.1 Study Design

The study involved two components: a descriptive analysis of influenza notifications in Queensland (tropical/subtropical climate) for the year 2022 compared with the five previous years (2017–2021) and a case–control study to estimate influenza VE against both laboratory‐confirmed influenza and influenza‐associated hospitalisation in Queensland in 2022.

In Queensland, confirmed influenza cases are notified based on laboratory definitive evidence [10]. Cases are electronically reported by public and private pathology laboratories to the Queensland Health Notifiable Conditions System (NoCS). In 2022, COVID‐19 was a reportable condition, notifiable on test request (polymerase chain reaction [PCR] and rapid antigen test [RAT]), and all test results, positive and negative, were reported to the NoCS.

### 2.2 Sources of Data

Data on laboratory‐confirmed influenza notifications were obtained from NoCS for the study period 2017–2022. Age‐specific midyear population figures for Queensland were obtained from the Australian Bureau of Statistics to calculate rates [11]. In 2022, the estimated population of Queensland was 5,322,058, of who 17% were aged 65 years and older and 6% were aged less than 5 years [11]. The Queensland Hospital Admitted Patient Data Collection data were routinely integrated with influenza‐associated hospitalisations captured in NoCS.

Laboratory‐confirmed influenza cases and COVID‐19 test (PCR) negative controls with unknown influenza testing status were included in the VE study. Specimen collection dates were from 1 May to 31 October 2022. The study design is similar to those with controls selected from the same population who are diagnosed for other diseases or not tested for influenza [9, 12]. COVID‐19 test controls who were also notified as influenza cases were excluded from the control population, which reduced the likelihood of misclassification.

Test results for cases and controls were extracted from NoCS and linked to the Australian Immunisation Register (AIR) to obtain history of influenza and COVID‐19 vaccination. Linkage was performed by the Data Services, Public Health Intelligence Branch, Queensland Health (see Supporting Information). For individuals with multiple negative COVID‐19 tests, only the first test during the study period was included. A sensitivity analysis was conducted using the pool of eligible controls that included individuals with positive COVID‐19 tests (see Supporting Information). The same exclusion criteria were applied for cases and controls (see Supporting Information).

#### 2.2.1 Matching

Controls were matched to cases on postcode, specimen collection dates (±14 days of COVID‐19 test specimen collection date from cases' specimen collection date) and age (controls drawn from the same age group as their case) (see Supporting Information).

Matched controls with the same date of birth and sex as their respective matched cases were excluded to avoid a case potentially being their own control. Controls that were matched to multiple cases were randomly assigned to one case. The maximum number of controls per case was restricted to 10. When more than 10 controls matched to an individual case, 10 controls were randomly selected.

Influenza cases recorded as being hospitalised in NoCS were included to estimate VE against influenza‐associated hospitalisation. An influenza case was reported as hospitalised if hospital admission was associated with influenza as a contributing factor or cause. These cases were matched to controls using the same criteria as that used for VE against laboratory‐confirmed influenza and VE estimated by age groups, dose completeness and number of doses received in 2022.

#### 2.2.2 Vaccination Status

Cases and controls were considered vaccinated if influenza vaccine/s were received in 2022. Cases who received influenza vaccine < 14 days prior to the specimen collection date were considered unvaccinated. The same criteria were applied to controls using the specimen collection date of their matched cases.

Among children 6 months to < 9 years of age, a second dose was considered valid if received ≥ 28 days after the first dose. For this cohort, complete vaccination status (completeness) referred to receipt of two doses in 2022 or odds ratio (OR), one dose in 2022 and one or more doses prior to 2022. Vaccination history from the AIR since 2017 was available for study participants.

### 2.3 Data Analysis

#### 2.3.1 Descriptive Epidemiology

Influenza notifications were analysed as counts of notified cases by the International Organization for Standardization (ISO) weeks of episode dates (earliest of onset and specimen collection dates) from 2017 to 2022. Notification rates per 100,000 population were calculated by year and age groups.

#### 2.3.2 VE

Crude ORs were calculated using conditional logistic regression. OR was adjusted for sex to calculate adjusted OR and estimate adjusted VE (VE_*adj*_). Crude VE, VE_*adj*_ and confidence intervals (CIs) were calculated for any dose received in 2022 for all age groups. For children < 9 years of age, vaccine completeness (no vaccine, complete, and incomplete course) and vaccine doses received in 2022 (none, one and two doses) were assessed. VE estimates were stratified by age group (see Supporting Information). Associations between overall vaccine uptake and case/control status were calculated using Pearson's Chi‐squared test. Data analyses were performed using Stata version SE 16.1 (StataCorp, College Station, TX, USA) and Microsoft Excel Version 2202 (Microsoft, Redmond, Washington).

Funded adjuvanted quadrivalent influenza vaccine coverage among vaccinated individuals aged ≥ 65 years was calculated as the total number of cases and controls who received adjuvanted quadrivalent influenza vaccine, divided by the total number of cases and controls included in the VE analysis who received any influenza vaccine.

### 2.4 Ethics and Other Approvals

Ethics approval for this project was obtained from the Queensland Health, Metro North Human Research Ethics Committee B (HREC/2022/MNHB/91287) and recognised by the Australian National University Human Research Ethics Committee. Approvals from the Australian Government Department of Health and Aged Care and Communicable Diseases Branch (CDB) (for AIR data usage) and the Queensland Health CDB Data Custodian and Public Health Act were also provided.

## 3 Results

### 3.1 Influenza Notifications and Demographic Characteristics, 2017–2022

**FIGURE 1:**
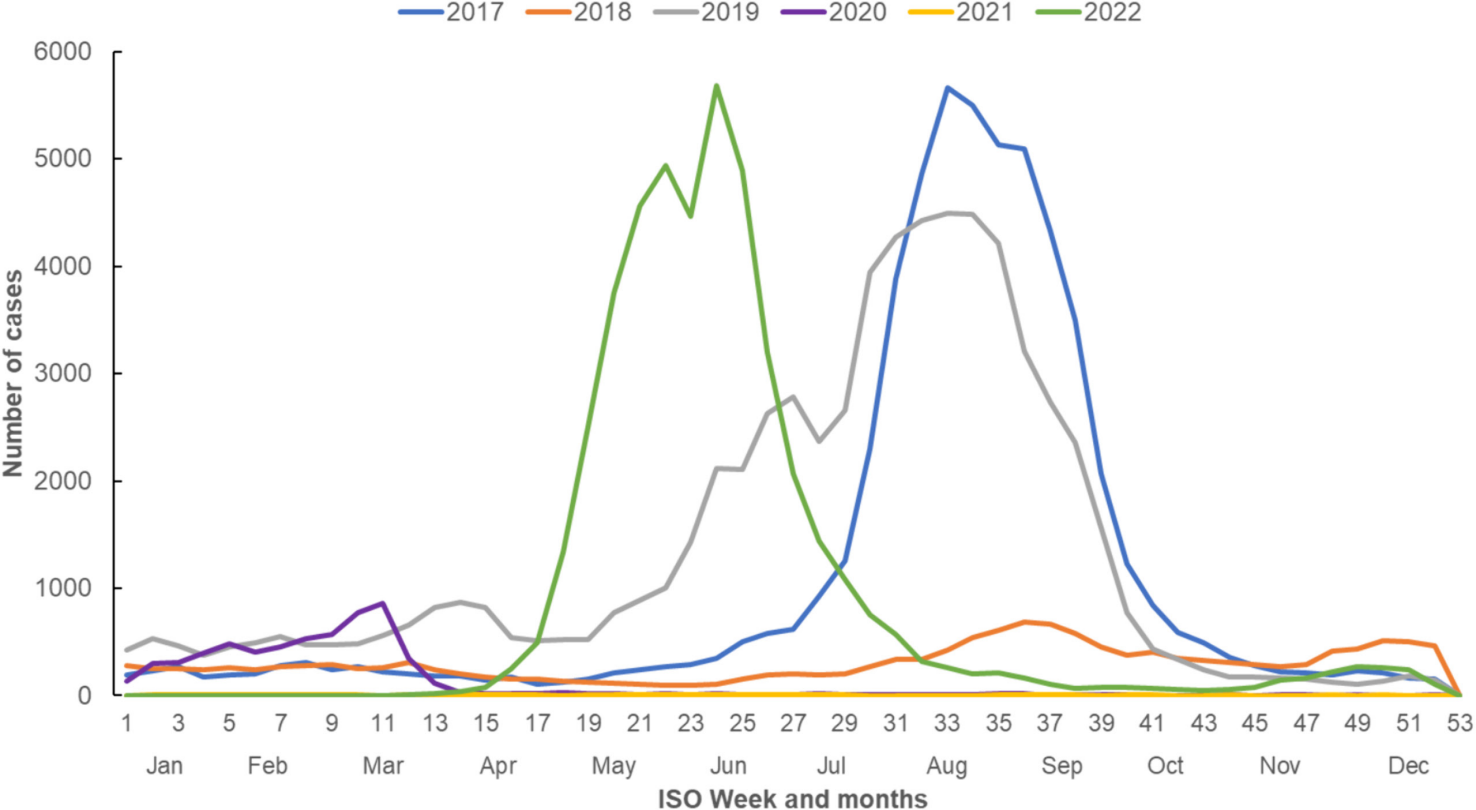
Laboratory‐confirmed influenza notifications by month and week of episode date, Queensland, 2017–2022.

A total of 45,311 laboratory‐confirmed influenza cases (confirmed by nucleic acid testing: *n* = 43,862, 96%) were notified in 2022 in Queensland (previous 5 years' annual average: 29,364). There were a higher number of cases in 2019 (*n* = 68,151) and 2017 (*n* = 56,614). Comparatively, smaller numbers of cases were notified in 2018 (*n* = 15,705) and during the COVID‐19 pandemic years, 2020 (*n* = 6047) and 2021 (*n* = 301). In 2022, the influenza season commenced in April–May, with notifications peaking in weeks 23–24 (June)—earlier compared to weeks 33–34 (August) in 2017 and 2019 (Figure 1).

**FIGURE 2:**
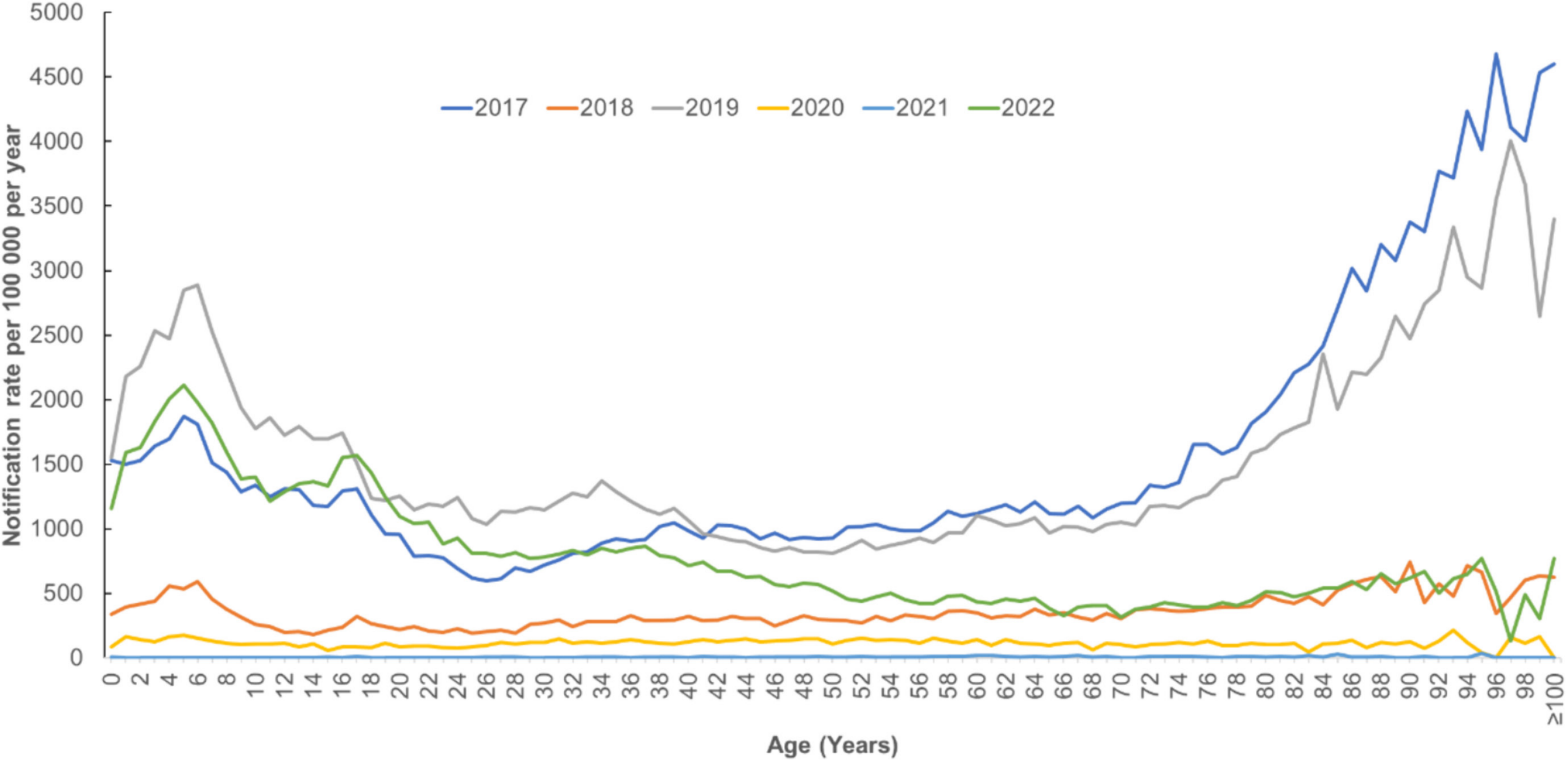
Laboratory‐confirmed influenza notification rate by year and age, Queensland, 2017–2022.

Influenza A predominated in 2022 (99.58%, *n* = 45,094) followed by influenza B (0.37%, *n* = 170), and dual infections with both A and B (0.04%, *n* = 16). Of the cases with influenza A subtypes known (*n* = 3883), 810 (20.86%) were influenza A H1, 3059 (78.77%) were influenza A H3 and 14 (0.36%) were dual infections with influenza A H1 and H3. Influenza notification rates were higher among children < 9 years old and older age groups (≥ 70 years) (Figure 2).

### 3.2 VE Against Laboratory‐Confirmed Influenza

#### 3.2.1 Case Control Matching

**FIGURE 3:**
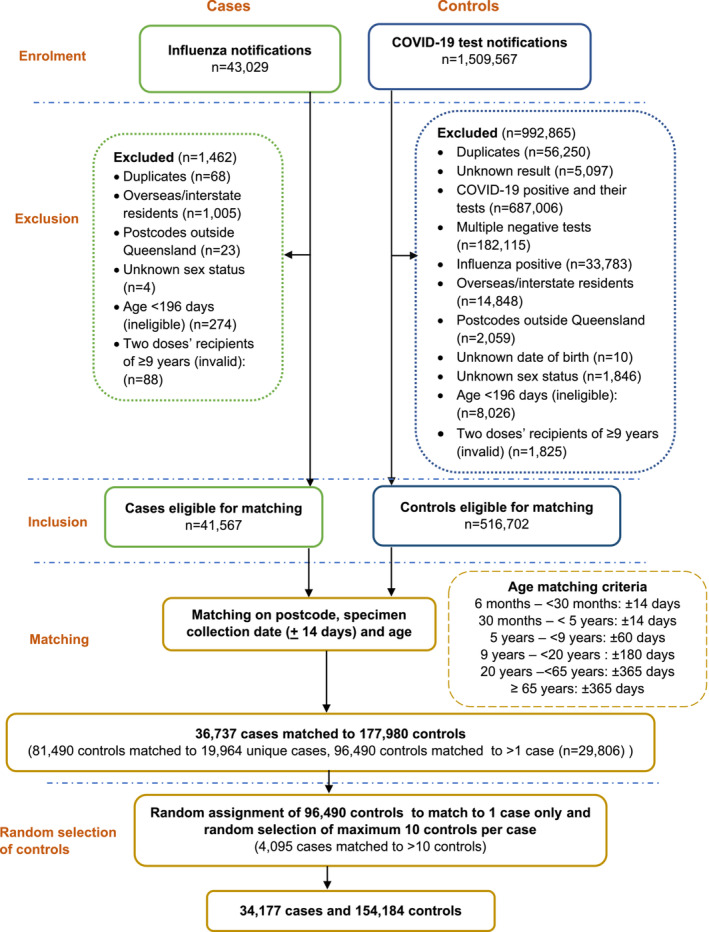
Case and control enrolment and matching on age, postcode, and specimen collection for VE estimates against laboratory‐confirmed influenza, 1 May 2022 to 31 October 2022.

There were 43,029 influenza notifications with a specimen collection date from 1 May to 31 October 2022 (Figure 3). Influenza cases (*n* = 41,567) and COVID‐19 negative controls (*n* = 516,702) were available for matching on age, postcode and specimen collection dates after applying exclusion criteria. A total of 154,184 controls matched to 34,177 cases were included in the analysis for estimation of VE. Of these, 14% of the cases were assigned 10 controls and 67% had 1–5 controls per case.

#### 3.2.2 Estimates of VE Against Laboratory‐Confirmed Influenza

The overall VE for receipt of any influenza vaccine preventing influenza notification among Queensland residents was 39% (95% CI: 36–41) (Table 1). The estimate was similar after adjusting for sex (VE_adj_) (Table 1). Influenza vaccine uptake was higher among controls (25.2%) than cases (15.4%) (*p* < 0.001). VE_adj_ was highest for children aged 30 months to < 5 years (61%, 95% CI: 49–70) and lowest for ≥ 65 years age groups (24%, 95% CI: 17–30). VE_adj_ against influenza A was 39% (95% CI: 37–41), with a similar point estimate for influenza B (32%, 95% CI ‐33–65). For those aged 65 years and older who were vaccinated (*n* = 10,807), 93% of cases (*n* = 1252) and controls (*n* = 8818) received funded adjuvanted quadrivalent vaccine. See Supporting Information for sensitivity analyses including COVID‐19 test positive and negative controls.

**TABLE 1:** Estimates of VE against laboratory‐confirmed influenza by vaccination status, age groups and influenza types, Queensland, 2022.

	Case *n* (%)	Control *n* (%)	Crude OR (95% CI)	Crude VE % (95% CI)	Adjusted VE% (95% CI)
Overall					
Not vaccinated	28,907 (84.6)	115,414 (74.8)	Ref.	Ref.	Ref.
Vaccinated	5270 (15.4)	38,770 (25.2)	0.61 (0.59–0.64)	39 (36–41)	39 (37–41)
6 to < 30 months					
Not vaccinated	1032 (90.6)	2371 (86.5)	Ref.	Ref.	Ref.
Vaccinated	107 (9.4)	370 (13.5)	0.63 (0.49–0.80)	37 (20–51)	37 (20–51)
30 months to < 5 years					
Not vaccinated	1272 (93.1)	2108 (85.8)	Ref.	Ref.	Ref.
Vaccinated	95 (6.9)	350 (14.2)	0.39 (0.30–0.51)	61 (49–70)	61 (49–70)
5–< 9 years					
Not vaccinated	2917 (93.5)	5985 (87.7)	Ref.	Ref.	Ref.
Vaccinated	201 (6.5)	838 (12.3)	0.46 (0.39–0.55)	54 (45–61)	54 (45–61)
≥ 9–< 20 years					
Not vaccinated	7216 (94.8)	20,156 (88.5)	Ref.	Ref.	Ref.
Vaccinated	397 (5.2)	2626 (11.5)	0.44 (0.39–0.49)	56 (51–61)	56 (51–61)
20–< 65 years					
Not vaccinated	14,822 (82.6)	76,180 (75.2)	Ref.	Ref.	Ref.
Vaccinated	3122 (17.4)	25,127 (24.8)	0.63 (0.61–0.66)	37 (34–39)	38 (34–40)
≥ 65 years					
Not vaccinated	1648 (55)	8614 (47.7)	Ref.	Ref.	Ref.
Vaccinated	1348 (45)	9459 (52.3)	0.77 (0.70–0.83)	23 (17–30)	24 (17–30)
Influenza A					
Not vaccinated	24,810 (84.6)	115,511 (74.9)	Ref.	Ref	Ref.
Vaccinated	5251 (15.4)	38,789 (24.1)	0.61 (0.59–0.64)	39 (36–41)	39 (37–41)
Influenza B					
Not vaccinated	47 (72.3)	144,274 (76.6)	Ref.	Ref.	Ref.
Vaccinated	18 (27.7)	44,022 (23.4)	0.66 (0.34–1.29)	34 (−29–66)	32 (−33–65)

*Note:* Vaccinated: Received at least one dose in 2022.

Abbreviations: OR, odds ratio; Ref., reference group; VE, vaccine effectiveness.

Assessing VE among children 6 months to < 9 years old by vaccination doses received in 2022 and in prior years, the point estimate for VE and VE_adj_ was higher for children who were completely vaccinated (55%, 95% CI: 49–61) compared to incompletely vaccinated (18%, 95% CI: −14–40) (Table 2).

**TABLE 2:** Estimates of VE against laboratory‐confirmed influenza among children, by vaccine completeness and doses, Queensland, 2022.

	Case *n* (%)	Control *n* (%)	Crude OR (95% CI)	Crude VE % (95% CI)	Adjusted VE% (95% CI)
Vaccine completeness					
6 months to < 9 years					
Not vaccinated	5221 (92.8)	10,464 (87.1)	Ref.	Ref.	Ref.
Incomplete	61 (1.1)	137 (1.1)	0.82 (0.60–1.14)	18 (−14–40)	18 (−14–40)
Complete	342 (6.1)	1421 (11.8)	0.45 (0.39–0.51)	55 (49–61)	55 (49–61)
6 to < 30 months					
Not vaccinated	1032 (90.6)	2371 (86.5)	Ref.	Ref.	Ref.
Incomplete	36 (3.2)	67 (2.4)	1.02 (0.66–1.59)	‐2 (−59–34)	‐2 (−59–35)
Complete	71 (6.2)	303 (11.1)	0.54 (0.41–0.71)	46 (29–59)	46 (29–59)
30 months to < 5 years					
Not vaccinated	1272 (93.1)	2108 (85.7)	Ref.	Ref.	Ref.
Incomplete	9 (0.6)	21 (0.9)	0.59 (0.26–1.35)	41 (−35–74)	41 (−36–74)
Complete	86 (6.3)	329 (13.4)	0.38 (0.29–0.50)	62 (50–71)	62 (50–71)
5–< 9 years					
Not vaccinated	2917 (93.6)	5985 (87.7)	Ref.	Ref.	Ref.
Incomplete	16 (0.5)	49 (0.7)	0.69 (0.38–1.25)	31 (−25–62)	31 (−25–62)
Complete	185 (5.9)	789 (11.6)	0.45 (0.37–0.53)	55 (47–63)	55 (47–63)
Vaccine doses received in 2022					
6 months–< 9 years					
Not vaccinated	5221 (92.8)	10,464 (87)	Ref.	Ref.	Ref.
One dose	389 (6.9)	1504 (12.5)	0.48 (0.42–0.54)	52 (46–58)	52 (46–58)
Two doses	14 (0.3)	54 (0.5)	0.54 (0.29–1.01)	46 (−1–71)	46 (−1–71)
6–< 30 months					
Not vaccinated	1032 (90.6)	2371 (86.5)	Ref.	Ref.	Ref.
One dose	95 (8.3)	322 (11.7)	0.64 (0.50–0.83)	36 (17–50)	36 (17–50)
Two doses	12 (1.1)	48 (1.8)	0.56 (0.28–1.11)	44 (−11–72)	44 (−11–72)
30 months to < 5 years					
Not vaccinated	1272 (93)	2108 (85.8)	Ref.	Ref.	Ref.
One dose	93 (6.8)	347 (14.1)	0.39 (0.30–0.50)	61 (50–70)	61 (50–70)
Two doses	2 (0.2)	3 (0.1)	0.78 (0.12–5.24)	22 (−424–88)	21 (−427–88)
5–< 9 years					
Not vaccinated	2917 (93.5)	5985 (87.7)	Ref.	Ref.	Ref.
One dose	201 (6.5)	835 (12.2)	0.46 (0.39–0.55)	54 (45–61)	54 (45–61)
Two doses	0	3 (0.1)	—	—	—

*Note:* Complete: two doses in 2022 or one dose in 2022 and at least one prior dose. Incomplete: one dose in 2022 and no prior dose.

Abbreviations: OR, odds ratio; Ref., reference group; VE, vaccine effectiveness.

### 3.3 Influenza VE Against Influenza‐Associated Hospitalisation

#### 3.3.1 Case Control Matching

Of the 3541 laboratory‐confirmed influenza notifications with a hospitalisation recorded, 3058 (86.3%) cases matched to 31,504 controls. After random selection and restriction of up to 10 controls per case, 3037 cases and 19,042 controls were used to estimate VE against influenza hospitalisation. Of these, 38% of the cases (*n* = 1161) had 10 controls assigned to them and 44% had 1–5 controls per case (See Figure S1).

#### 3.3.2 Estimates of Influenza VE Against a Notified Case Being Hospitalised

Overall, VE and VE_adj_ by sex against hospitalisation due to influenza was 54% (95% CI: 48–59) (Table 3). Vaccine uptake was higher among controls (28.6%) compared to cases (16.5%) (*p* < 0.001). When stratified by age group, VE_adj_ was highest among those aged 5–< 9 years (84%, 95% CI: 48–95).

**TABLE 3:** Estimates of VE against influenza‐associated hospitalisation by vaccination status, age group, and influenza types, Queensland, 2022.

	Case *n* (%)	Control *n* (%)	Crude OR (95% CI)	Crude VE% (95% CI)	VE_adj_ % (95% CI)
Overall					
Not vaccinated	2537 (83.5)	13,589 (71.4)	Ref.	Ref.	Ref.
Vaccinated	500 (16.5)	5453 (28.6)	0.46 (0.41–0.52)	54 (48–59)	54 (48–59)
6 to < 30 months					
Not vaccinated	141 (94.6)	383 (92.7)	Ref.	Ref.	Ref.
Vaccinated	8 (5.4)	30 (7.3)	0.57 (0.24–1.34)	43 (−34–76)	44 (−33–77)
30 months to < 5 years					
Not vaccinated	118 (92.9)	240 (90.6)	Ref.	Ref.	Ref.
Vaccinated	9 (7.1)	25 (9.4)	0.60 (0.24–1.47)	40 (−47–76)	41 (−46–76)
5–< 9 years					
Not vaccinated	166 (98.2)	526 (89.6)	Ref.	Ref.	Ref.
Vaccinated	3 (1.8)	62 (10.5)	0.16 (0.05–0.52)	84 (49–95)	84 (48–95)
≥ 9–< 20 years					
Not vaccinated	297 (96.4)	1649 (91.5)	Ref.	Ref.	Ref.
Vaccinated	11 (3.6)	153 (8.5)	0.42 (0.22–0.79)	58 (21–78)	59 (21–78)
20–< 65 years					
Not vaccinated	1217 (88.7)	7899 (77.2)	Ref.	Ref.	Ref.
Vaccinated	155 (11.3)	2331 (22.8)	0.40 (0.33–0.48)	60 (52–67)	61 (53–68)
≥ 65 years					
Not vaccinated	598 (65.6)	2892 (50.4)	Ref.	Ref.	Ref.
Vaccinated	314 (34.4)	2852 (49.6)	0.53 (0.46–0.62)	47 (38–54)	47 (38–54)
Influenza types					
Influenza A					
Not vaccinated	2528 (83.5)	13,598 (71.4)	Ref.	Ref.	Ref.
Vaccinated	500 (16.5)	5453 (28.6)	0.47 (0.42–0.52)	53 (48–58)	54 (48–59)
Influenza B					
Not vaccinated	3 (100)	16,123 (73)	Ref.	Ref.	Ref.
Vaccinated	0	5953 (27)	—	—	—

*Note:* Vaccinated: Received at least one dose in 2022.

Abbreviations: OR, odds ratio; Ref., reference group; VE_adj_, VE adjusted by sex; VE, vaccine effectiveness.

Assessing VE against hospitalisation by completeness for children aged 6 months to < 9 years, a high proportion of hospitalised cases and matched controls were unvaccinated (Table 4). The point estimates of effectiveness of complete doses compared to incomplete dose were higher among all age groups of children, except 30 months to < 5 years old.

**TABLE 4:** Estimates of vaccine effectiveness against influenza‐associated hospitalisation among children, by vaccine completeness and doses, Queensland, 2022.

	Case *n* (%)	Control *n* (%)	Crude OR (95% CI)	Crude VE % (95% CI)	VE_adj_ % (95% CI)
Vaccine completeness					
6 months to < 9 years					
Not vaccinated	425 (95.5)	1149 (90.7)		Ref.	Ref.
Incomplete	6 (1.4)	16 (1.3)	0.82 (0.28–2.42)	18 (−142–72)	17 (−146–72)
Complete	14 (3.1)	101 (8)	0.33 (0.18–0.61)	67 (39–82)	67 (39–82)
6–< 30 months					
Not vaccinated	141 (94.6)	383 (92.7)		Ref.	Ref.
Incomplete	4 (2.7)	6 (1.5)	1.30 (0.30–5.68)	−30 (−468–70)	−37 (−496–69)
Complete	4 (2.7)	24 (5.8)	0.40 (0.13–1.19)	60 (−19–87)	62 (−15–87)
30 months to < 5 years					
Not vaccinated	118 (92.9)	240 (90.6)		Ref.	Ref.
Incomplete	1 (0.8)	3 (1.1)	0.50 (0.04–5.81)	50 (−468–70)	49 (−480–96)
Complete	8 (6.3)	22 (8.3)	0.61 (0.24–1.53)	39 (−19–87)	40 (−53–76)
5–< 9 years					
Not vaccinated	166 (98.2)	526 (89.5)		Ref.	Ref.
Incomplete	1 (0.6)	7 (1.2)	0.60 (0.07–5)	40 (−400–93)	40 (−404–93)
Complete	2 (1.2)	55 (9.3)	0.12 (0.03–0.49)	88 (51–97)	89 (52–97)
Vaccine doses received in 2022					
6 months to < 9 years					
Not vaccinated	425 (95.5)	1149 (90.8)	Ref.	Ref.	Ref.
One dose	20 (4.5)	113 (8.9)	0.40 (0.24–0.69)	60 (31–76)	60 (31–76)
Two doses	0	4(0.3)	—		
6–< 30 months					
Not vaccinated	141 (94.6)	383 (92.7)	Ref.	Ref.	Ref.
One dose	8 (5.4)	26 (6.3)	0.63 (0.26–1.50)	37 (−50–74)	37 (−50–74)
Two doses	0	4 (1)	—		
30 months to < 5 years					
Not vaccinated	118 (92.9)	240 (90.6)	Ref.	Ref.	Ref.
One dose	9 (7.1)	25 (9.4)	0.60 (0.24–1.47)	40 (−47–76)	40 (−47–76)
Two doses	—	—	—	—	—
5–< 9 years					
Not vaccinated	3 (1.8)	62 (10.5)	Ref.	Ref.	Ref.
One dose	166 (98.2)	526 (89.5)	0.16 (0.05–0.52)	84 (48–95)	84 (48–95)
Two doses	—	—	—	—	—

*Note:* Complete: two doses in 2022 or one dose in 2022 and at least one prior dose. Incomplete: one dose in 2022 and no prior dose.

Abbreviations: OR, odds ratio; Ref., reference group; VE_adj_, VE adjusted by sex; VE, vaccine effectiveness.

## 4 Discussion

In Queensland, the influenza season started earlier in 2022 than the five preceeding years. Total influenza notification counts returned to frequencies similar to the pre‐COVID‐19 period; however, the number of cases peaked earlier in the season. Following public funding of free influenza vaccine for Queensland residents in 2022, the overall VE of any dose of vaccine was estimated to be 39% against laboratory‐confirmed influenza. The majority of the cases included in our study were due to influenza A (99.7%), and thus, VE was similar against this influenza virus type. VE against laboratory‐confirmed influenza was lower for children 6 to < 30 months (37%) compared to 30 months to < 5 years of age (61%). The opportunity for this youngest cohort to have previously been exposed to influenza viruses was very small, with limited virus circulation during their lives (2020–2021) [6]. The older cohort of children also had a greater opportunity to receive complete vaccine doses and be exposed to circulating virus in earlier years. As expected, VE decreased with increasing age and was lowest for those ≥ 65 years old.

Overall, VE against influenza‐associated hospitalisation (54%) was higher than VE against laboratory‐confirmed influenza (39%). VE against hospitalisation was highest for children aged 5–< 9 years (84%). This cohort likely had substantial exposure to circulating virus in previous seasons, with the notification rate high in this age cohort in 2019. VE among children < 9 years of age with complete doses and one dose in 2022 was also observed to be higher in comparison to incomplete doses recipients. This reinforces our previous fjndings and the Australian Immunisation Handbook recommendation that children < 9 years should be encouraged to receive the recommended complete age and history relevant dose course of influenza vaccine [9, 13]. Our VE estimates among children were in similar range as previoulsy reported in Queensland [9]. Previous Australian studies estimated VE with a range of 40%–79% against influenza‐associated hospitalisation [14, 15, 16, 17, 18, 19, 20]. These estimates are expected to differ depending on the match between vaccine compositions, circulating influenza virus, geographical locations, and study designs, period and population.

We found that 93% of individuals aged ≥ 65 years received the recommended adjuvanted vaccine funded for this age group. Despite this, VE in this age group appears low (24%) for preventing infection in the H3N2 dominated year, with VE against hospitalisation higher at 47%. VE against infection in H3N2 dominated seasons was similarly low in Europe and the United States in 2021–2022 [21, 22, 23]. However, those findings appear to be due to mismatch between the circulating strain and vaccine seen in the Northern Hemisphere in the 2021–2022 season [21, 22]. In Australia, strains circulating in 2022 were characterised as antigenically similar to the corresponding vaccine components for almost all of the tested specimens, including for 94.5% of H3N2 isolates assessed [24]. Further work on more effective vaccines in older age groups, particularly against H3 viruses, is required.

This study has a number of strengths. Vaccination history was obtained from the AIR by data linkage with laboratory‐confirmed influenza notifications. Reporting of NIP vaccines to AIR was mandatory from 1 July 2021 for Australian vaccine providers [25]. Underreporting, to the extent if does occur, is likely to be similar in cases and controls due to the same source of vaccination data and the absence of outcome (influenza infection) at the time of reporting. We did not have data on individual laboratory test types for influenza cases in our dataset; however, the substantial majority (96%) of all notified cases were diagnosed or confirmed by PCR testing.

As with all observational studies, the reported effect estimates could be biassed by confounding factors, including access to health care facilities for laboratory testing and immunisation and due to differences in demographic characteristics (sex, age and location) between cases and controls. Any difference in demographic characteristics is likely to have been accounted for through the matching process, while differences due to access to healthcare facilities are likely to have been minimised by the availability of publicly funded diagnostic testing and influenza vaccine for all Queenslanders. All controls were tested for COVID‐19, which is likely to reduce differences between cases and controls in terms of healthcare access and health‐seeking behaviour. This inclusion criterion reduces the possibility of controls having influenza but not being tested and consequently is likely to minimise misclassification bias.

Influenza notification rates were lower in older adults in 2022, compared to 2019 and 2021. The lower notification rates in older adults may be due to COVID‐19 mitigation measures and higher vaccination coverage [26]. Influenza vaccine coverage among adults aged 65 years and older was also higher in 2022 (67.5%–73%) for this age group compared to previous years (e.g., 54.7%–61.5% in 2019) [27]. Lower rates of infection among the older age group would have minimal effect in the VE estimates, as an adequate number of cases were matched to controls for the analysis.

Previous studies have shown that including those who test positive for COVID‐19 as controls can underestimate influenza VE due to the correlation of COVID‐19 vaccination and influenza vaccination behaviours [28, 29]. Individuals who are less likely to be vaccinated against COVID‐19 are also unlikely to be vaccinated against influenza compared to the source population [29, 30]. Our sensitivity analysis showed that including COVID‐19 test positive controls reduced overall VE (see Supporting Information). We addressed this issue of confounding bias by restricting our control group to include those who had negative COVID‐19 tests only [28, 29].

The impact of being able to use AIR data to undertake this study cannot be understated. It is only since 2021 that Australian jurisdictions have had access to their own AIR data for the purposes of undertaking public health related activities [31]. For this work, we were able to use AIR to assess the immunisation status of cases and controls, minimising misclassification and associated bias. Using AIR in a similar way in the future will make routine and automated VE assessments possible and more timely, adding further confidence and certainty to our recommendations around the use of influenza vaccine each year.

## 5 Conclusion

In Queensland, seasonal influenza activity commenced earlier in 2022 than the previous years. Influenza vaccines provided varying levels of protection against laboratory‐confirmed influenza among different age groups, which was driven by dose completeness in those < 9 years of age, possibly due to natural immunity development due to prior virus exposure, and multifactorial lower effectiveness in older people. However, the level of protection against hospitalisation was moderate in all age groups, which highlighted the significance of funded influenza vaccine in reducing severe disease outcomes.

## Author Contributions

**Ashish C. Shrestha:** conceptualization, methodology, formal analysis, writing – original draft, visualization, investigation. **Emma Field:** conceptualization, methodology, writing – review and editing, supervision, investigation. **Dharshi Thangarajah:** formal analysis, writing – review and editing. **Ross Andrews:** methodology, writing – review and editing. **Robert S. Ware:** conceptualization, methodology, writing – review and editing. **Stephen B. Lambert:** conceptualization, methodology, supervision, writing – review and editing, project administration, resources, investigation.

## Conflicts of Interest

The authors declare no conflicts of interest.

### Peer Review

The peer review history for this article is available at https://www.webofscience.com/api/gateway/wos/peer‐review/10.1111/irv.70007.

## Supporting information


**Data S1.** Supporting Information.

**Figure S1.** Case and control enrolment and matching on age, postcode, and specimen collection for VE estimates against influenza‐associated hospitalisation, 1 May 2022 to 31 October 2022.

**Table S1.** Case and control age matching criteria.
**Table S2.** Estimates of VE against laboratory‐confirmed influenza including positive and negative COVID‐19 tests and stratified by test results vs controls with negative COVID‐19 test only (primary analysis), Queensland, 2022.

## Data Availability

Research data are not shared.
