# Supplementary material for: Influenza Epidemiology and Vaccine Effectiveness Following Funded Influenza Vaccine in Queensland, Australia, 2022
Source: Influenza Other Respir Viruses. 2024 Sep 25;18(9):e70007. doi: 10.1111/irv.70007 (PMC11423337; doi:10.1111/irv.70007)
Supplement: Supplementary file 1 — Data S1. Supporting Information. [file IRV-18-e70007-s002.docx]

**Supporting Information**

**1. Data linkage for influenza vaccination status**

Linkage keys were generated based on the matching criteria in the following order between Notifiable conditions System (NoCS) and Australian Immunisation Register (AIR). If matching with the first linkage key was unsuccessful, then the second linkage key was used and so on.

1. Medicare number (8 digits) AND first name AND date of birth
2. Last name AND first name AND date of birth AND postcode
3. Medicare number (8 digits) AND similar string sounds of first name AND similar string sounds of last name AND date of birth
4. Similar string sounds of first name AND date of birth AND last name AND postcode

**2. Exclusion criteria**

For both influenza and COVID-19, tests with the same notification dates, notifications with residence postcodes outside Queensland, unknown sex, and unknown age were excluded. Cases and controls were restricted to children aged at least 6 months and 14 days (≥196 days) at specimen collection date, to account for the 14 days required to confer active immunity following vaccination. Individuals aged ≥9 years who received two or more doses of vaccine in 2022 were excluded. COVID-19 test controls who were notified as influenza cases during the study period were also excluded from the control population.

**3. Case and control age matching criteria**

The age matching criteria were as follows, with controls drawn from the same age-group as their case (Supporting Information Table 1).

**4. Sensitivity analysis**

**4.1. Using COVID-19 positive and negative controls**

A sensitivity analysis was also performed including individuals with both positive and negative COVID-19 tests as controls and matched on postcode, age, and specimen collection date. This provided 37,600 cases (positive COVID-19: n=5,230, negative COVID-19: n=29,558, no COVID-19 test: n=2,812) and 246,376 controls (positive COVID-19: n=136,990, negative COVID-19: n= 109,386) for the analysis. The overall VE_adj_ against laboratory-confirmed influenza was 36% (95% CI: 36–38) and after stratification by COVID-19 test results, VE_adj_ for negative COVID-19 controls (40%, 95% CI 37–42) was higher than for for positive COVID-19 controls (23%, 95% CI 16–30) (Supporting Information Table 2).

**5. Figure of case and control enrolment and matching for VE estimates against influenza associated hospitalisation** (Supporting Information Figure 1)
