# Supplementary material for: Influenza Epidemiology and Vaccine Effectiveness Following Funded Influenza Vaccine in Queensland, Australia, 2022
Source: Influenza Other Respir Viruses. 2024 Sep 25;18(9):e70007. doi: 10.1111/irv.70007 (PMC11423337; doi:10.1111/irv.70007)
Supplement: Supplementary file 2 — Figure S1. Case and control enrolment and matching on age, postcode, and specimen collection for VE estimates against influenza‐associated hospitalisation, 1 May 2022 to 31 October 2022. [file IRV-18-e70007-s003.pdf]

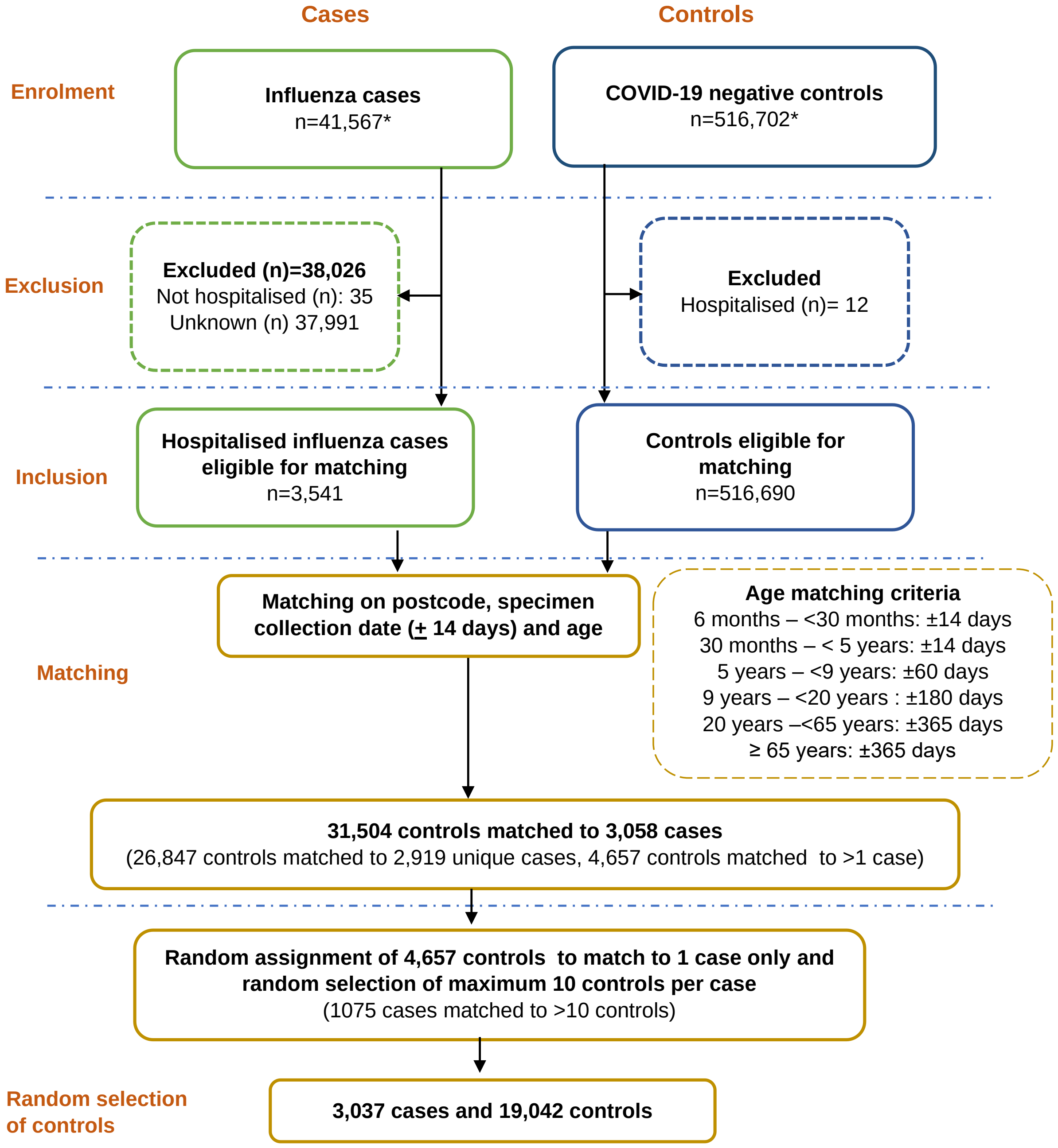

**Number of cases and controls before matching included in VE estimates against laboratory-confirmed influenza*
