# Supplementary material for: Influenza Epidemiology and Vaccine Effectiveness Following Funded Influenza Vaccine in Queensland, Australia, 2022
Source: Influenza Other Respir Viruses. 2024 Sep 25;18(9):e70007. doi: 10.1111/irv.70007 (PMC11423337; doi:10.1111/irv.70007)
Supplement: Supplementary file 3 — Table S1. Case and control age matching criteria. Table S2. Estimates of VE against laboratory‐confirmed influenza including positive and negative COVID‐19 tests and stratified by test results vs controls with negative COVID‐19 test only (primary analysis), Queensland, 2022. [file IRV-18-e70007-s001.docx]

**Table 1.** Case and control age matching criteria

| **Age-groups of cases and controls** | **Difference in days** |
| --- | --- |
| 6 months–<30 months | ±14 days |
| 30 months–<5 years | ±14 days |
| 5 years–<9 years | ±60 days |
| 9 years–<20 years | ±180 days |
| 20 years–<65 years | ±365 days |
| ≥65 years | ±365 days |

**Table 2.** Estimates of VE against laboratory-confirmed influenza including positive and negative COVID-19 tests and stratified by test results vs controls with negative COVID-19 test only (primary analysis), Queensland, 2022

|  | **Case n (%)** | **Control n (%)** | **Crude OR**  **(95% CI)** | **Crude VE %**  **(95% CI)** | **VE_adj_**  **(95% CI)** |
| --- | --- | --- | --- | --- | --- |
| ***COVID-19 positive and negative controls matched to cases*** | | | | | |
| *Not vaccinated* | 32,024 (85.2) | 190,565 (77.3) | *Ref.* | *Ref.* | *Ref.* |
| *Vaccinated* | 5,576 (14.8) | 55,811 (22.7) | 0.64 (0.62–0.66) | 36 (34–38) | 36 (36–-38) |
| ***COVID test results^†^*** | | | | | |
| ***COVID-19 positive*** | | | | | |
| *Not vaccinated* | 4,353 (83.2) | 107,142 (78.2) | *Ref.* | *Ref.* | *Ref.* |
| *Vaccinated* | 877 (16.8) | 29,848 (21.8) | 0.79 (0.72–0.87) | 21 (13–28) | 23 (16–30) |
| ***COVID-19 negative*** | | | | | |
| *Not vaccinated* | 25,127 (85) | 83,423 (76.3) | *Ref.* | *Ref.* | *Ref.* |
| *Vaccinated* | 4,431 (15) | 25,963 (23.7) | 0.61 (0.58–0.63) | 39 (37–42) | 40 (37–42) |
| ***COVID-19 negative controls only matched to cases (Primary analysis)*** | | | | | |
| *Not vaccinated* | 28,907 (84.6) | 115,414 (74.8) | *Ref.* | *Ref.* | *Ref.* |
| *Vaccinated* | 5,270 (15.4) | 38,770 (25.2) | 0.61 (0.59–0.64) | 39 (36–41) | 39 (37–41) |

*VE: Vaccine effectiveness; VE_adj_: VE adjusted by sex; Ref: Reference group;* ***^†^****COVID-19 positive and negative tests included in controls and stratified by test result*
